# Relationship between corticosteroid use and incidence of ventilator-associated pneumonia in COVID-19 patients: a retrospective multicenter study

**DOI:** 10.1186/s13054-022-04170-2

**Published:** 2022-09-27

**Authors:** Ouriel Saura, Anahita Rouzé, Ignacio Martin-Loeches, Pedro Povoa, Louis Kreitmann, Antoni Torres, Matthieu Metzelard, Damien Du Cheyron, Fabien Lambiotte, Fabienne Tamion, Marie Labruyere, Claire Boulle Geronimi, Charles-Edouard Luyt, Martine Nyunga, Olivier Pouly, Arnaud W. Thille, Bruno Megarbane, Anastasia Saade, Eleni Magira, Jean-François Llitjos, Iliana Ioannidou, Alexandre Pierre, Jean Reignier, Denis Garot, Jean-Luc Baudel, Guillaume Voiriot, Gaëtan Plantefeve, Elise Morawiec, Pierre Asfar, Alexandre Boyer, Armand Mekontso-Dessap, Fotini Bardaka, Emili Diaz, Christophe Vinsonneau, Pierre-Edouard Floch, Nicolas Weiss, Adrian Ceccato, Antonio Artigas, David Nora, Alain Duhamel, Julien Labreuche, Saad Nseir, coVAPid Study Group, Mathilde Bouchereau, Sean Boyd, Luis Coelho, Julien Maizel, Pierre Cuchet, Wafa Zarrougui, Déborah Boyer, Jean-Pierre Quenot, Mehdi Imouloudene, Marc Pineton de Chambrun, Thierry Van der Linden, François Arrive, Sebastian Voicu, Elie Azoulay, Edgard Moglia, Frédéric Pene, Catia Cilloniz, Didier Thevenin, Charlotte Larrat, Laurent Argaud, Bertrand Guidet, Damien Contou, Alexandra Beurton, David Meguerditchian, Keyvan Razazi, Vassiliki Tsolaki, Mehdi Marzouk, Guillaume Brunin, Clémence Marois, Luis Morales

**Affiliations:** 1 Médecine Intensive-Réanimation, CHU de Lille, 59000 Lille, France; 2 INSERM U1285, Université de Lille, CNRS, UMR 8576 – UGSF – Unité de Glycobiologie Structurale et Fonctionnelle, 59000 Lille, France; 3 Department of Intensive Care Medicine, Multidisciplinary Intensive Care Research Organization (MICRO), St. James’s Hospital, Dublin, Ireland; 4 Department of Clinical Medicine, School of Medicine, Trinity College Dublin, Dublin, Ireland; 5 Hospital Clinic, IDIBAPS, Universidad de Barcelona, Ciberes, Barcelona, Spain; 6 Polyvalent Intensive Care Unit, Hospital de São Francisco Xavier, CHLO, Lisbon, Portugal; 7 NOVA Medical School, CHRC, New University of Lisbon, Lisbon, Portugal; 8 Center for Clinical Epidemiology and Research Unit of Clinical Epidemiology, OUH Odense University Hospital, Odense, Denmark; 9 Critical Care Department, Hospital Universitari Parc Tauli, Sabadell, Spain; 10 Departament de Medicina, Universitat Autonoma de Barcelona, Barcelona, Spain; 11 Service de Médecine Intensive Réanimation, CHU Amiens Picardie, 80000 Amiens, France; 12 Department of Pulmonology, Hospital Clinic of Barcelona, University of Barcelona, IDIBAPS, CIBERES, Barcelona, Spain; 13 Department of Medical Intensive Care, Caen University Hospital, 14000 Caen, France; 14 Service de Réanimation Polyvalente, Centre Hospitalier de Valenciennes, Valenciennes, France; 15 Medical Intensive Care Unit, Rouen University Hospital, UNIROUEN, Inserm U1096, FHU- REMOD-VHF, 76000 Rouen, France; 16 Department of Intensive Care, François Mitterrand University Hospital, Dijon, France; 17 Service de Réanimation et de Soins Intensifs, Centre Hospitalier de Douai, Douai, France; 18 Service de Médecine Intensive Réanimation, Institut de Cardiologie, Groupe Hospitalier Pitié-Salpêtrière, Assistance Publique – Hôpitaux de Paris, Paris Cedex 13, France; 19 Service de Réanimation, Centre Hospitalier de Roubaix, Roubaix, France; 20 Service de Médecine Intensive Réanimation, Hôpital Saint Philibert GHICL, Université Catholique, Lille, France; 21 CHU de Poitiers, Médecine Intensive Réanimation, CIC 1402 ALIVE, Université de Poitiers, Poitiers, France; 22 Department of Medical and Toxicological Critical Care, Lariboisière Hospital, INSERM UMRS-1144, Paris Cité University, Paris, France; 23 Service de Médecine Intensive Réanimation, Hôpital Saint-Louis, 75010 Paris, France; 24 National and Kapodistrian University of Athens, Evangelismos Hospital, Athens, Greece; 25 Medical Intensive Care Unit, Cochin Hospital, Assistance Publique – Hôpitaux de Paris, Paris, France; 26 First Department of Pulmonary Medicine and Intensive Care Unit, National and Kapodistrian University of Athens, Sotiria Chest Hospital, Athens, Greece; 27 Service de Réanimation Polyvalente, Centre Hospitalier de Lens, Lens, France; 28 Service de Médecine Intensive Réanimation, CHU de Nantes, Nantes, France; 29 Service de Médecine Intensive Réanimation, CHU de Tours, Hôpital Bretonneau, 37044 Tours Cedex 9, France; 30 Service de Médecine Intensive - Réanimation, Hôpital Edouard Herriot, Hospices Civils de Lyon, 69437 Lyon Cedex 03, France; 31 Service de Médecine Intensive Réanimation, Hôpital Saint-Antoine, Assistance Publique-Hôpitaux de Paris, 75012 Paris, France; 32 Sorbonne Université, Assistance Publique-Hôpitaux de Paris, Service de Médecine Intensive Réanimation, Hôpital Tenon, Paris, France; 33 Service de Réanimation Polyvalente, CH Victor Dupouy, Argenteuil, France; 34 Service de Médecine Intensive-Réanimation et Pneumologie, Assistance Publique-Hôpitaux de Paris, Hôpital Pitié Salpêtrière, Paris, France; 35 Sorbonne Université, Inserm UMRS Neurophysiologie Respiratoire Expérimentale et Clinique, Assistance Publique-Hôpitaux de Paris, Hôpital Pitié Salpêtrière, Paris, France; 36 Département de Médecine Intensive Réanimation, CHU d’Angers, 49933 Angers Cedex 9, France; 37 Service de Médecine Intensive Réanimation, CHU de Bordeaux, 33000 Bordeaux, France; 38 Assistance Publique-Hôpitaux de Paris, Hôpitaux Universitaires Henri-Mondor, Service de Médecine Intensive Réanimation, 94010 Créteil, France; 39 CARMAS, Université Paris Est Créteil, 94010 Créteil, France; 40 INSERM U955, Institut Mondor de Recherche Biomédicale, 94010 Créteil, France; 41 Intensive Care Unit, University Hospital of Larissa, University of Thessaly, Biopolis, 41110 Larissa, Greece; 42 Intensive Care Unit, Hôpital de Béthune, 62408 Béthune, France; 43 Service de Réanimation, Hôpital Duchenne, 62200 Boulogne-sur-Mer, France; 44 Département de Neurologie, Unité de Médecine Intensive Réanimation Neurologique, Sorbonne Université, Assistance Publique-Hôpitaux de Paris, Hôpital de la Pitié-Salpêtrière, Paris, France; 45 Brain Liver Pitié-Salpêtrière (BLIPS) Study Group, INSERM UMR_S 938, Centre de Recherche Saint-Antoine, Maladies Métaboliques, Biliaires et Fibro-Inflammatoire du Foie, Institute of Cardiometabolism and Nutrition (ICAN), Sorbonne Université, Paris, France; 46 Groupe de Recherche Clinique en Réanimation et Soins intensifs du Patient en Insuffisance Respiratoire aiguE (GRC-RESPIRE), Sorbonne Université, Paris, France; 47 Intensive Care Unit, Hospital Universitari Sagrat Cor, Grupo Quironsalud, Barcelona, Spain; 48 Critical Care Center, Corporacion Sanitaria Universitaria Parc Tauli, CIBER Enfermedades Respiratorias, Autonomous University of Barcelona, Sabadell, Spain; 49 Univ. Lille, ULR 2694-METRICS: Evaluation des Technologies de Santé et des Pratiques Médicales, 59000 Lille, France; 50 Biostatistics Department, CHU de Lille, 59000 Lille, France

**Keywords:** SARS-CoV-2, COVID-19, Ventilator-associated lower respiratory tract infections, Corticosteroids

## Abstract

**Background:**

Ventilator-associated pneumonia (VAP) is common in patients with severe SARS-CoV-2 pneumonia. The aim of this ancillary analysis of the coVAPid multicenter observational retrospective study is to assess the relationship between adjuvant corticosteroid use and the incidence of VAP.

**Methods:**

Planned ancillary analysis of a multicenter retrospective European cohort in 36 ICUs. Adult patients receiving invasive mechanical ventilation for more than 48 h for SARS-CoV-2 pneumonia were consecutively included between February and May 2020. VAP diagnosis required strict definition with clinical, radiological and quantitative microbiological confirmation. We assessed the association of VAP with corticosteroid treatment using univariate and multivariate cause-specific Cox’s proportional hazard models with adjustment on pre-specified confounders.

**Results:**

Among the 545 included patients, 191 (35%) received corticosteroids. The proportional hazard assumption for the effect of corticosteroids on the incidence of VAP could not be accepted, indicating that this effect varied during ICU stay. We found a non-significant lower risk of VAP for corticosteroid-treated patients during the first days in the ICU and an increased risk for longer ICU stay. By modeling the effect of corticosteroids with time-dependent coefficients, the association between corticosteroids and the incidence of VAP was not significant (overall effect *p* = 0.082), with time-dependent hazard ratios (95% confidence interval) of 0.47 (0.17–1.31) at day 2, 0.95 (0.63–1.42) at day 7, 1.48 (1.01–2.16) at day 14 and 1.94 (1.09–3.46) at day 21.

**Conclusions:**

No significant association was found between adjuvant corticosteroid treatment and the incidence of VAP, although a time-varying effect of corticosteroids was identified along the 28-day follow-up.

**Supplementary Information:**

The online version contains supplementary material available at 10.1186/s13054-022-04170-2.

## Introduction

The novel severe acute respiratory syndrome coronavirus 2 (SARS-CoV-2) can lead to a severe respiratory tract infection (coronavirus disease 2019 (COVID-19)) and hit the world with multiple pandemic waves from December 2020. During the first surge of COVID-19, studies reported that around 80% of patients admitted to hospital for COVID-19 would require oxygen support [1, 2] and a high rate of them an invasive mechanical ventilation (IMV) due to acute respiratory distress syndrome (ARDS) leading to high mortality rates [3, 4]. IMV during ARDS exposes patients to severe complications, such as ventilator-associated pneumonia (VAP).

Several studies have recently described the high incidence of ventilator-associated lower respiratory tract infection (VA-LRTI) in COVID-19 patients ranging from 30 to 84% [4–7]. Previous studies demonstrated a significantly higher incidence of VA-LRTI and notably VAP in SARS-CoV-2 pneumonia patients *versus* non-SARS-CoV-2 pneumonia patients [5, 8–10]. High rates of ARDS, alveolar inflammation, prolonged IMV, lung microbiota alteration, COVID-19-related specific lesions, neuromuscular blocking and immunosuppressive agent use could explain this high rate of VAP in SARS-CoV-2 pneumonia patients [11, 12].

The positive results of the randomized controlled multicenter trial RECOVERY [13] and its further confirmation in large meta-analysis [14] have placed dexamethasone as the first line agent for treating hospitalized COVID-19 patients with a significantly improved 28-day survival, especially in the subgroup of patients invasively ventilated. Yet, the impact of such treatment on the incidence of VAP in COVID-19 patients is still a matter for debate, as available data are scarce and conflicting [3, 15–19].

Hence, we sought to determine the relationship between adjuvant corticosteroid treatment and the incidence of VAP in a large cohort of COVID-19 patients invasively ventilated for more than 48 h, during the first surge of the SARS-CoV-2 pandemic. Our hypothesis was that VAP incidence would be higher in corticosteroid-treated *versus* non-treated COVID-19 patients.

The primary objective was to compare the incidence of VAP in patients receiving or not adjuvant corticosteroid treatment. Secondary objectives were to determine the relationship between adjuvant corticosteroid treatment and 28-day mortality, duration of mechanical ventilation and ICU length of stay, based on the occurrence of VAP. We also evaluated microbiology of VAP in both groups of patients.

## Methods

### Study design and population

This is a planned ancillary analysis of the coVAPid study, which design was previously described [8]. Briefly, it was a multicenter retrospective observational cohort conducted in 36 ICUs in Europe (28 centers in France, 3 in Spain, 3 in Greece, 1 in Portugal and 1 in Ireland) aimed to determine the relationship between SARS-CoV-2 pneumonia as compared to influenza pneumonia or no viral infection and the incidence of VA-LRTI. The present analysis was restricted to the population of SARS-CoV-2 pneumonia. Patients were 18-year-old or above and received IMV for more than 48 h. Patients were excluded if data related to corticosteroid treatment were not available.

Patients were consecutively included in each center starting from the beginning of the COVID-19 pandemic if they had a positive polymerase chain reaction (PCR) test for the SARS-CoV-2 virus on nasopharyngeal swab or respiratory secretions.

The Ethics Committee and Institutional Review Boards approved the coVAPid study protocol (Comité de Protection des Personnes Ouest VI; approved by April 14, 2020; registration number RIPH:20.04.09.60039) as minimal-risk research using data collected for routine clinical practice. The requirement for informed consent was waived, and patients or their relative received information about the study and were given the possibility to refuse the use of their personal data. The coVAPid database was registered into the “Commission Nationale de l’Informatique et des Libertés” in accordance with the French law. The coVAPid study was registered at ClinicalTrials.gov, number NCT04359793.

Demographic characteristics, severity scores and comorbidities, past medical history and ongoing treatments, prior hospitalization and exposition to antibiotics (during the past three months) were collected for each patient at baseline. During hospitalization, clinical, biological and radiological findings were recorded as well as the different treatments received during ICU stay (i.e., prone positioning, extra corporeal membrane oxygenation (ECMO)). Antibiotic and corticosteroid use was recorded as type, time of initiation, duration of exposure before occurrence of VAP, maximum daily dose expressed as prednisone equivalent dose (for corticosteroid treatment).

### Objectives and outcomes

Our primary objective was to determine the impact of corticosteroid use on the incidence of VAP in SARS-CoV-2 pneumonia patients. The primary outcome was the occurrence of VAP [8, 20]. Briefly, new or progressive infiltrates on chest X-rays and two of the following criteria were needed for the diagnosis of VAP: (1) leucocyte count greater than 12,000 cells per μL or less than 4000 cells per μL, (2) hyperthermia above 38.5 °C or hypothermia under 36.5 °C, (3) purulent tracheal secretions. Microbiological confirmation was required to confirm the diagnosis, with at least 10^5^ colony-forming units (CFUs) per mL for endotracheal aspirates or 10^4^ CFU per mL for bronchoalveolar lavage. VAP episodes were prospectively recorded and confirmed by two distinct physicians as well as the chest X-rays readings. Only first VAP episodes were taken into account.

The secondary objective was to determine the impact of adjuvant corticosteroid treatment on 28-day mortality, MV duration and ICU length of stay.

### Statistical analysis

Categorical variables are reported as absolute number and percentage, whereas continuous variables are expressed as median with interquartile range (25th–75th percentile). Patient characteristics at ICU admission and during ICU stay were described according to the use or not of corticosteroids without statistical comparisons. The 28-day cumulative incidence of VAP, extubation alive and ICU discharge alive were estimated using the Kalbfleisch and Prentice method [21], considering extubation within 28-day (dead or alive), death under MV, or death during ICU as a competing events. The 28-day cumulative incidence of all-cause mortality was estimated using the Kaplan–Meier method.

The association between corticosteroids and VAP was assessed using Cox regression with cause-specific hazard function considering initiation of corticosteroid treatment as a time-varying covariate. In this model, extubation within 28 days (dead or alive) without VAP was considered as a competing event. The corticosteroid time-varying variable was coded 0 during the period before the start of the treatment and 1 from the day of initiation of corticosteroids until the occurrence of VAP or MV withdrawal or death within the 28-day period of follow-up. The cause-specific hazard ratios (cHR) for corticosteroid vs. non-corticosteroid exposure were calculated as effect size. Hazard assumption proportionality of corticosteroids during follow-up was assessed by using the scaled Schoenfeld residuals plots. Since the assumption was not satisfied, the corticosteroids effect was modeled using time-dependent coefficient by including corticosteroids and corticosteroids*time interaction terms as covariates into cause-specific Cox’s models and the overall corticosteroids effect was assessed by the likelihood ratio test.

Similarly, we investigated the association of corticosteroids with patient’s outcomes censored at day-28 (overall survival, MV duration, length of ICU stay) by using a Cox’s regression model with cause-specific hazard for MV duration (considering as event of interest extubation alive and death under MV as competing event) and for length of ICU stay (considering as event of interest ICU discharge alive, and death in ICU as competing event) [22]. Since there was no deviation on proportionality assumption, the corticosteroid effect was only modeled as a time-varying covariate without time-dependent coefficients.

In addition, we investigated the effect of the occurrence of VAP on the association between corticosteroid use and these outcomes by including in the models the VAP covariate and the corticosteroids x VAP interaction (both treated as time-varying covariates).

All associations were further investigated after adjustment for pre-specified confounders already known to be associated with VAP and patients’ outcomes (age, gender, BMI, SAPS II, MacCabe classification, immunosuppression, recent hospitalization, recent antibiotics, shock, ARDS and cardiac arrest). To avoid case-deletion in multivariate analyses due to presence of missing data in covariates, multivariable Cox’s models were performed after handling missing data by using a multiple imputation procedure [23]. Specifically, imputation of missing values was performed using a regression-switching approach (chained equations with m = 20 obtained) under the “missing at random” assumption, using all baseline characteristics (see Table 1) and outcomes, with a predictive mean matching method for quantitative variables and a logistic regression model (binary, ordinal or multinomial) for categorical variables. Estimates obtained in the different imputed data sets were combined using Rubin’s rules [24]. Sensitivity analysis on complete cases (patients without missing data on covariates) was also performed.

**Table 1:** Patient characteristics at ICU admission

	No corticosteroids (*n *= 354)	Corticosteroids (*n *= 191)
Age, years	64 (53–72)	65 (57–72)
Men	256 (72.3)	134 (70.2)
BMI (Kg/m)*	28.4 (25.4–33.0)	29.6 (26.9–34.3)
Severity scores		
SAPS II†	41 (32–54)	43 (33–60)
SOFA Score‡	6 (3–8)	7 (3–9)
Comorbidity scores		
MacCabe classification		
Non-fatal	296/336 (88.1)	157/186 (85.3)
Fatal < 5 years	36/336 (10.7)	25/186 (13.6)
Fatal < 1 year	4/336 (1.2)	2/186 (1.1)
Charlson comorbidity index^§^	3 (1–4)	3 (2–4)
Chronic diseases		
Diabetes mellitus	109/353 (30.9)	52/189 (27.5)
Chronic renal failure	17/349 (4.9)	15/187 (8.0)
Heart disease	70/351 (19.9)	30/187 (16.0)
Chronic heart failure	13/350 (3.7)	8/186 (4.3)
COPD	19/350 (5.4)	18/188 (9.6)
Chronic respiratory failure	15/350 (4.3)	5/186 (2.7)
Cirrhosis	4/350 (1.1)	4/187 (2.1)
Immunosuppression	25/350 (7.1)	27/187 (14.4)
Active smoking	17/350 (4.9)	11/188 (5.9)
Alcohol abuse	23/349 (6.6)	10/187 (5.3)
Location before ICU admission		
Home	163/354 (46.0)	95/191 (49.7)
Hospital ward	136/354 (38.4)	70/191 (36.6)
Another ICU	55/354 (15.5)	26/191 (13.6)
Recent hospitalization (< 3 months)	24/354 (6.8)	18/189 (9.5)
Recent antibiotics (< 3 months)	45/354 (12.7)	26/190 (13.7)
Causes for ICU admission		
Shock	63/348 (18.1)	39/187 (20.9)
Acute respiratory failure	327/354 (92.4)	172/190 (90.5)
ARDS	244/352 (69.3)	125/188 (66.5)
Neurological failure	14/344 (4.1)	12/182 (6.6)
Cardiac arrest	2/343 (0.6)	1/182 (0.5)
Acute renal failure	58/344(16.9)	37/182 (20.3)

Values are as no./No.(%) or median (interquartile range). * 31 missing values (n = 10 in corticosteroid group); † 42 missing values (*n * = 17 in corticosteroid group); ‡ 21 missing values (*n* = 8 in corticosteroid group); § 19 missing values (*n *=  8 in corticosteroid group)

McCabe classification of comorbidities and likelihood of survival, likely to survive > 5 years, 1–5 years, < 1 year; Chronic kidney disease, KDOQI CKD classification stage 4 or 5 (creatinine clearance < 30 ml/mn); Chronic heart failure, NYHA class III or IV; Heart disease, ischemic heart disease or atrial fibrillation; Cirrhosis, Child–Pugh score B or C; Immunosuppression if hematological malignancy, allogeneic stem cell transplant, solid cancer, organ transplant, HIV or immunosuppressive drugs; More than one cause for ICU admission is possible

*ARDS* Acute respiratory distress syndrome, *COPD* chronic obstructive pulmonary disease, *ICU* intensive care unit, *SAPS II* Simplified Acute Physiology Score II, *SOFA* Sequential Organ Failure Assessment

Statistical testing was performed with a two-tailed α level of 0.05. Data were analyzed using the SAS software package, release 9.4 (SAS Institute, Cary, NC). Statistical analysis is fully detailed in the online data supplement.

## Results

**Fig. 1:**
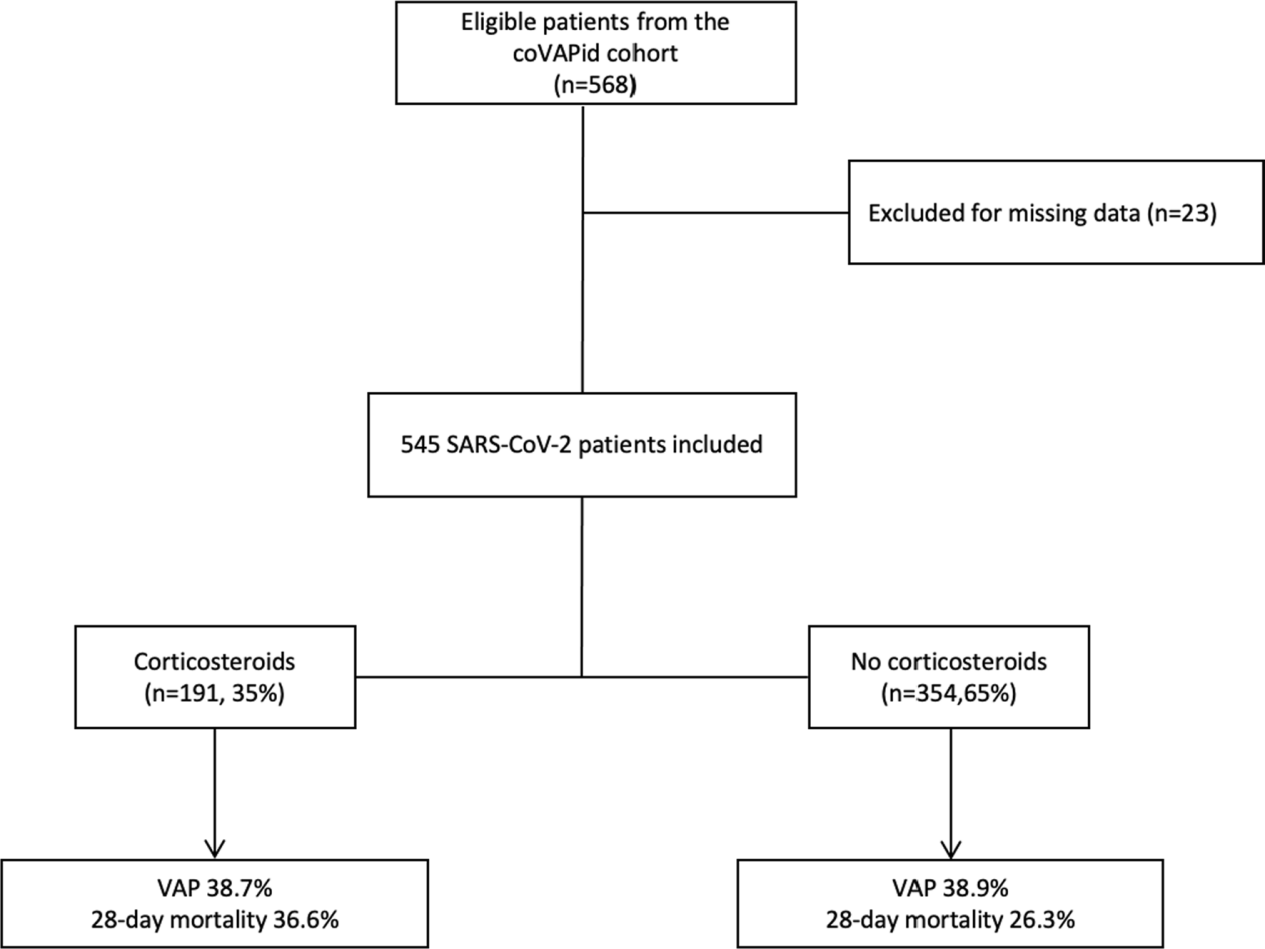
Study flowchart

From March 2020 through May 2020, 568 patients receiving IMV > 48 h for SARS-CoV-2 pneumonia were eligible for this study. Twenty-three patients (4%) were excluded due to missing data. Among the 545 included patients, 191 (35%) received corticosteroids (Fig. 1).

### Patient characteristics at ICU admission

At ICU admission, the majority of patients were men with a median age of 64 year-old in corticosteroid and no-corticosteroid groups. Body mass index (BMI), SAPS II and SOFA scores, recent hospitalization (< 3 months) and recent antibiotic use (< 3 months) were comparable between both groups. In the corticosteroid group, the percentage of patients with immunosuppression was higher than in patients who did not receive corticosteroids. The main causes for ICU admission were acute respiratory failure and ARDS in both groups (Table 1).

### Patient characteristics during ICU stay

Patient characteristics during ICU stay are presented in Table 2. Over 90% of patients in both groups had an antibiotic treatment during their ICU stay with a slightly longer duration of treatment in the corticosteroid group. Prone positioning was more frequent in patients who received corticosteroids than in those who did not. ECMO use was comparable in the two groups. 28-day mortality was higher in patients who received corticosteroids than in those who did not. Duration of MV and ICU length of stay were longer in the corticosteroid group than in the no-corticosteroid group.

**Table 2:** Patient characteristics during ICU stay

	No corticosteroids (*n* = 354)	Corticosteroids (*n* = 191)
Corticosteroids		
Hydrocortisone	–	52/187 (27.8)
Dexamethasone	–	46/187 (24.6)
Methylprednisolone	–	90/187 (48.1)
Highest daily dose, mg	–	100 (50–133)
Exposure period, days	–	6 (3–8)
Antibiotic treatment	308/328 (93.9)	177/182 (97.3)
Duration, days	12 (7–18)	14 (9–20)
Prone positioning	228/353 (64.6)	143/191 (74.9)
ECMO	39/354 (11.0)	22/190 (11.6)
28-day outcomes		
MV duration, days	14 (8–22)	17 (10–25)
28-day mortality	93/354 (26.3)	70/191 (36.6)
ICU length of stay, days	17 (11–27)	20 (13–28)

Values are as no./No.(%) or median (interquartile range)

Highest daily dose of corticosteroids is reported as prednisone equivalent. Exposure period is defined from the day of initiation of corticosteroids until the occurrence of VAP or MV withdrawal or death within the 28-day of follow-up

*ECMO* extracorporeal membrane oxygenation, *ICU* intensive care unit, *MV* mechanical ventilation

In the corticosteroid group, patients mainly received methylprednisolone (48.1%) for a median time of 6 days (3–8 days) before occurrence of VAP. The highest daily dose of prednisone equivalent was 100 mg (50–133 mg).

### Incidence of VAP and corticosteroid use

In the corticosteroid group, 74 (38.7%) patients developed at least one episode of VAP *versus* 138 (38.9%) patients in the no-corticosteroid group. As shown in Additional file 1: e-Fig. 1, the proportional hazard assumption for the effect of corticosteroids on the incidence of VAP, assessed by the scaled Schoenfeld residuals, revealed a time tendency indicating that this effect varied during ICU stay. By modeling the effect of corticosteroids with time-dependent coefficients, the association of corticosteroids with incidence of VAP was not significant before and after pre-specified adjustment (Table 3), with time-dependent adjusted hazard ratios (95% confidence interval) of 0.47 (0.17–1.31) at day 2, 0.95 (0.63–1.42) at day 7, 1.48 (1.01–2.16) at day 14 and 1.94 (1.09–3.46) at day 21. Similar results were found in sensitivity complete case analysis. Additional file 1: e-Table 1 depicts the association of different corticosteroids with VAP in all study patients.

**Table 3:** Association between corticosteroid treatment use and outcomes in all study patients

	Unadjusted analysis		Adjusted analysis^b^			
			Multiple imputation analysis^c^		Complete case-analysis	
	cHR (95%CI)	*P* Value	cHR (95%CI)	*P* Value	cHR (95%CI)	*P* Value
VAP						
Overall effect		0.13^a^		0.082^a^		0.056^a^
At day 2	0.43 (0.15–1.17)		0.47 (0.17–1.31)		0.43 (0.14–1.24)	
At day 7	0.86 (0.58–1.28)		0.95 (0.63–1.42)		0.95 (0.63–1.44)	
At day 14	1.34 (0.92–1.94)		1.48 (1.01–2.16)		1.59 (1.05–2.38)	
At day 21	1.75 (0.99–3.08)		1.94 (1.09–3.46)		2.16 (1.16–4.02)	
28-day mortality	2.01 (1.46–2.75)	< 0.0001	1.67 (1.20–2.33)	0.002	1.47 (1.02–2.10)	0.034
MV duration	1.14 (0.89–1.45)	0.30	1.25 (1.01–1.54)	0.036	1.18 (0.88–1.56)	0.25
Length of ICU stay	0.71 (0.54–0.95)	0.017	1.00 (0.80–1.24)	0.99	0.77 (0.56–1.07)	0.12

*ARDS* acute respiratory distress syndrome, *BMI* body mass index, *cHR* cause-specific hazard ratio, *CI* confidence interval, *ICU* intensive care unit, *SAPS II* Simplified Acute Physiology Score II, *VAP* ventilator-associated pneumonia

^a^*P* Value for the effect of corticosteroids assessed by including corticosteroids and time*corticosteroids terms into Cox’s regression model to account the violation of proportional hazard assumption

^b^Adjusted for age, sex, BMI, SAPS II, MacCabe classification, immunosuppression, recent hospitalization, recent antibiotics, shock, ARDS, cardiac arrest (^c^after handling missing values by multiple imputation (*m* = 20)

### Patient characteristics at the day of VAP and microbiological data

VAP episodes were mainly diagnosed with endotracheal aspirates in both groups. SOFA score, modified CPIS and PaO2/FiO2 ratio did not differ at the day of VAP between the two groups. Thirty-five (25%) patients had at least one VAP recurrence in the no-corticosteroids group *versus* 8 (11%) in the corticosteroids group. A higher percentage of patients received antibiotic treatment at the day of VAP in the corticosteroid group *versus* no-corticosteroid group (Table 4).

**Table 4:** Description of patients at the day of VAP diagnosis

	No corticosteroids (*n* = 138)	Corticosteroids (*n* = 74)
Total number of VAP		
1	103/138 (74.6)	66/74 (89.2)
2	29/138 (21.0)	6/74 (8.1)
3	5/138 (3.6)	1/74 (1.4)
4	1/138 (0.7)	1/74 (1.4)
5	0/138 (0.0)	1/74 (1.4)
SOFA score^a^	8 (5–11)	8 (5–11)
Diagnostic procedure		
Endotracheal aspirates	83/137 (60.6)	47/73 (64.4)
Bronchoalveolar lavage	54/137 (39.4)	26/73 (35.6)
Modified CPIS^a^	6 (5–7)	6 (4–7)
PaO_2_/FiO_2_^b^	130 (91–180)	139 (95–188)
Antibiotic treatment	122/138 (88.4)	71/74 (95.9)
Appropriate antibiotic treatment	94/133 (70.7)	48/73 (65.8)

Values are as no./No.(%) or median (interquartile range)

*VAP* ventilator-associated pneumonia, *SOFA* Sequential Organ Failure Assessment, *CPIS* clinical pulmonary infection score

^a^8 missing values (1 in corticosteroids group)

^b^13 missing values (3 in corticosteroids group)

Gram-negative bacilli were the main bacteria responsible for VAP in the 2 groups (60%). *Pseudomonas aeruginosa,* Enterobacter spp. and *Escherichia coli* were the most frequently identified bacteria. A Higher proportion of VAP due to multidrug-resistant bacteria (MDR) was found in the corticosteroid-treated group (Table 5).

**Table 5:** Microorganisms responsible for VAP

	No corticosteroids (*n* = 138)	Corticosteroids (*n* = 74)
Gram-positive cocci	22 (15.9)	13 (17.6)
MSSA	12 (8.8)	7 (9.5)
MRSA	5 (3.6)	0 (0.0)
Enterococcus spp.	3 (2.2)	4 (5.4)
*Streptococcus pneumoniae*	2 (1.5)	2 (2.7)
Gram-negative bacilli	83 (60)	44 (59.5)
*Pseudomonas aeruginosa*	24 (17.5)	17 (23)
*Enterobacter* spp.	20 (14.6)	14 (18.9)
*Klebsiella pneumoniae*	6 (4.4)	5 (6.8)
*Escherichia coli*	12 (8.8)	2 (2.7)
*Acinetobacter baumannii*	9 (6.6)	2 (2.7)
*Stenotrophomonas maltophilia*	2 (1.5)	0 (0.0)
*Serratia marcescens*	2 (1.5)	2 (2.7)
*Citrobacter freundii*	4 (2.9)	0 (0.0)
*Proteus mirabilis*	1 (0.7)	1 (1.4)
*Haemophilus influenza*	2 (1.5)	0 (0.0)
*Morganella morganii*	1 (0.7)	1 (1.4)
Other*	17 (12.3)	9 (12.1)
Polymicrobial	15 (10.9)	8 (10.8)
Multidrug-resistant isolates	26 (19.0)	21 (28.4)

*VAP* Ventilator-associated pneumonia

Values are as no. (%)

Missing values n = 1 (in the no-corticosteroid group)

MRSA, methicillin-resistant *Staphylococcus aureus*; MSSA, methicillin-sensitive *Staphylococcus aureus*

^*^Contains other *Streptococcus* Spp., other *Klebsiella* Spp., other *Citrobacter* Spp. and other bacteria

### Impact of corticosteroid use on patient outcomes

Twenty-eight-day mortality was significantly associated with corticosteroid exposure in unadjusted analysis and after adjusting for pre-specified confounders in complete case analysis, and after multiple imputation analysis. In adjusted analysis, corticosteroid use was associated with prolonged duration of MV after multiple imputation analysis, but not with ICU length of stay (Table 3). Associations between different corticosteroids and VAP, 28-day mortality, MV duration and ICU length of stay are presented in e-Table 1

VAP occurrence did not significantly modify the relationship between corticosteroids and mortality, duration of MV, or length of ICU stay (Table 6). However, a significant association was found between corticosteroids exposure and ICU mortality in the absence of exposure to VAP. Twenty-eight-day cumulative incidence of VAP, all-cause mortality, extubation alive and ICU discharge alive are presented in Additional file 1: e-Fig. 2.

**Table 6:** Association between corticosteroid use and mortality, MV duration and ICU length of stay according to VAP

	Unadjusted analysis			Adjusted analysis^a^					
				Multiple imputation analysis^b^			Complete case-analysis		
	cHR (95%CI)	*P* Value	*P* Het	cHR (95%CI)	*P* Value	*P* Het	cHR (95%CI)	*P* Value	*P* Het
All-cause 28-day mortality									
No VAP	2.36 (1.60–3.46)	< 0.0001	0.18	1.95 (1.31–2.90)	0.001	0.15	1.61 (1.03–2.51)	0.034	0.44
VAP	1.50 (0.87–2.59)	0.14		1.18 (0.66–2.10)	0.56		1.21 (0.67–2.17)	0.51	
MV duration									
No VAP	1.20 (0.90–1.61)	0.27	0.40	1.29 (0.96–1.73)	0.086	0.19	1.46 (1.03–2.05)	0.03	0.050
VAP	0.96 (0.62–1.50)	0.68		0.90 (0.57–1.42)	0.66		0.81 (0.49–1.31)	0.39	
Length of ICU stay									
No VAP	0.68 (0.48–0.96)	0.027	0.82	0.76 (0.53–1.07)	0.11	0.78	0.84 (0.56–1.26)	0.40	0.46
VAP	0.73 (0.44–1.19)	0.21		0.69 (0.42–1.14)	0.15		0.65 (0.38–1.12)	0.12	

*ARDS* acute respiratory distress syndrome, *BMI* body mass index, *cHR* cause-specific hazard ratio, *CI* confidence interval, *ICU* intensive care unit *SAPS II* Simplified Acute Physiology Score II, *VAP* ventilator-associated pneumonia

^a^Adjusted for age, gender, BMI, SAPS II, McCabe classification, immunosuppression, recent hospitalization, recent antibiotics, shock, ARDS, cardiac arrest

^b^After handling missing values by multiple imputation (*m* = 20)

*P* Het: *P* Value for the heterogeneity test of effects according to occurrence of VAP

## Discussion

In this ancillary study of the coVAPid study, we found no significant association between adjuvant corticosteroid treatment and the incidence of VAP in a population of SARS-CoV-2 patients invasively ventilated for over 48 h. However, we found a time-varying effect of corticosteroid adjuvant therapy on the incidence of VAP over the 28-day period.

The incidence of VAP in the SARS-CoV-2 population was high, nearly half of the patients presenting at least one VAP episode during ICU stay, irrespective of corticosteroid use. This result is in line with several previous studies that reported high incidence of VAP in critically ill COVID-19 patients [3, 8–10, 17].

Following the RECOVERY trial, corticosteroid adjuvant use dramatically increased in critically ill COVID-19 patients, although only 15% patients were invasively ventilated in this trial. In invasively ventilated COVID-19 patients, high rates of ARDS, prolonged ICU stay and IMV duration along with immunologic disorders and neuromuscular blocking agent use expose these patients to higher risk of developing VAP and one could question the impact of wide use of corticosteroids on the incidence of hospital-acquired infections (HAI) and VAP in this population, already at higher risk for these infections.

In COVID-19 patients, VAP is a serious complication, as our group and others demonstrated a significant association between VAP and mortality in this population [25, 26]. To date, there is no prospective interventional study that evaluated the impact of corticosteroid use on the incidence of VAP, as the RECOVERY study itself did not include it as an outcome. However, there is a growing body of evidence in the literature pointing out the increased risk of developing VAP in COVID-19 patients treated with corticosteroids [16–19, 27, 28].

A recent study by Mesland et al. [17]*.* reported, among 322 COVID-19 patients invasively ventilated, a significantly higher incidence for VA-LRTI in patients receiving corticosteroid adjuvant therapy compared with those who did not. Corticosteroids were found to be an independent factor associated with VA-LRTI in a multivariable adjusted analysis. Another recent study found the same association between corticosteroid use and VA-LRTI [18]. However, other studies suggested no association between corticosteroid exposure and the incidence of VA-LRTI in COVID-19 [15, 29].

In our study, we did not find a significant association between corticosteroid exposure and VAP incidence during the 28-day period of follow-up. However, our results suggest a trend toward a time-varying effect of corticosteroid exposure on the incidence of VAP. This time-varying effect increased all along the 28-day follow-up, and the association between corticosteroid use and VAP became statistically significant after the 14th day of mechanical ventilation. Several explanations could be provided for this time-varying effect. First, corticosteroid-immunosuppressive effect is a time-dependent but also a cumulative-dose-dependent mechanism [30] that may explain the late occurrence of hospital-acquired infections in treated patients. Second, sepsis-associated immunosuppression is a time-dependent process occurring in the late course of ICU stay, resulting in immunoparalysis and ICU-acquired infection [31, 32]. Finally, SARS-CoV-2-mediated immune dysfunction is associated with a progressive lymphopenia [33, 34], and a multifactorial immunosuppression culminating after day 14 [35] that exposes patients to late-onset ICU-acquired infection.

Our study reports higher 28-day mortality and longer MV duration in the group of patients who received adjuvant corticosteroid treatment, as compared to those who did not. The main reason for this discrepancy with results of randomized controlled trials [13, 36] might be the observational design of this study taking place before the RECOVERY era when corticosteroid prescriptions were at the discretion of attending physicians who selected the sickest patients to receive these treatments. Septic shock, non-resolving ARDS and intense systemic inflammation were the main reasons for corticosteroid adjuvant therapy, and this led de facto to a poorer prognosis in the corticosteroid-treated patients. Several other observational studies also failed to demonstrate this benefit from corticosteroids [17, 36].

Microorganisms responsible for VAP in our study did not differ between the corticosteroid treated and non-treated patients. In line with previous results, Gram-negative bacilli were the main bacteria found in respiratory tract samples with a high rate of Enterobacteriaceae and *Pseudomonas aeruginosa.* Methicillin-sensitive *Staphylococcus aureus* was the main Gram-positive cocci, and the rate of multidrug-resistant bacteria was also high [3, 15, 17].

To our knowledge, our study is the largest multicenter study to examine the impact of corticosteroid adjuvant therapy on the incidence of VAP in COVID-19 patients, and the first to show a varying association between corticosteroid use and the incidence of VAP. Additional strengths of our study are the large number of patients, the multicenter design and the strict definition of VAP with quantitative microbiological documentation for each event. However, some limitations should be mentioned. First, the observational retrospective design of our study taking place before the RECOVERY era is responsible for possible selection bias with disparate use of corticosteroids among centers that intentionally selected the sickest patients to receive corticosteroids, leading to poorer outcomes. Second, a small proportion of included patients (30%) received corticosteroids, which could also introduce a selection bias. Third, in our study we collected all steroid prescriptions irrespective of variations in molecule, duration and dosage. The impact of this discrepancy on patient outcomes might be real, yet no data support this in the literature.

## Conclusions

We found no significant association between corticosteroid adjuvant therapy and the incidence of VAP in the population of invasively ventilated patients for a SARS-CoV-2 pneumonia. However, we found a time-varying effect of corticosteroid adjuvant therapy on the cause-specific estimated risk of developing VAP throughout patient course in ICU. Further studies are needed to clarify the role and effects of adjuvant corticosteroid treatment in the sickest COVID-19 patients. Future research should also investigate the interest of a corticosteroid-use strategy based on patient inflammatory phenotypes.

## Supplementary Information

**Additional file 1.** Supplementary e-figures and e-tables.

## Data Availability

All data needed to evaluate the conclusions in this article are present and tabulated in the main text or the appendix. This article is the result of an original retrospective cohort. For individual de-identified raw data that underlie the results reported in this article, please contact the corresponding authors.

## Abbreviations

ARDS: Acute respiratory distress syndrome
BMI: Body mass index
BSI: Bloodstream infection
CFU: Colony-forming unit
CPIS: Clinical pulmonary infection score
COVID-19: Coronavirus disease 2019
ECMO: Extra corporeal membrane oxygenation
HAI: Hospital-acquired infection
ICU: Intensive care unit
IMV: Invasive mechanical ventilation
PCR: Polymerase chain reaction
SAPS II: Simplified Acute Physiology Score II
SARS-CoV-2: Severe acute respiratory syndrome coronavirus 2019
SOFA: Sequential Organ Failure Assessment
VA-LRTI: Ventilator-associated lower respiratory tract infection
VAP: Ventilator-associated pneumonia
VAT: Ventilator-associated tracheobronchitis
