# Supplementary material for: Relationship between corticosteroid use and incidence of ventilator-associated pneumonia in COVID-19 patients: a retrospective multicenter study
Source: Crit Care. 2022 Sep 27;26:292. doi: 10.1186/s13054-022-04170-2 (PMC9513297; doi:10.1186/s13054-022-04170-2)
Supplement: Supplementary file 1 — Additional file 1. Supplementary e-figures and e-tables. [file 13054_2022_4170_MOESM1_ESM.docx]

**Online Supplementary data**

**Results**

Among the 569 patients eligible for the coVAPid original cohort, 568 were included. One patient was excluded because of SARS-CoV-2 and influenza co-infection.

**e-figure 1: Association of corticosteroid treatment and VAP across time assessed by Schoenfeld residual plots.**

This plot represent the effect (regression coefficient) of corticosteroid use on the risk of VAP across time. The horizontal line corresponds to no association, and values under the horizontal lines corresponds to a protective association while values upper the horizontal lines corresponds to detrimental association. We observed a gradual increase in regression coefficient with time, with P-value for proportional hazard assumption reported.

^
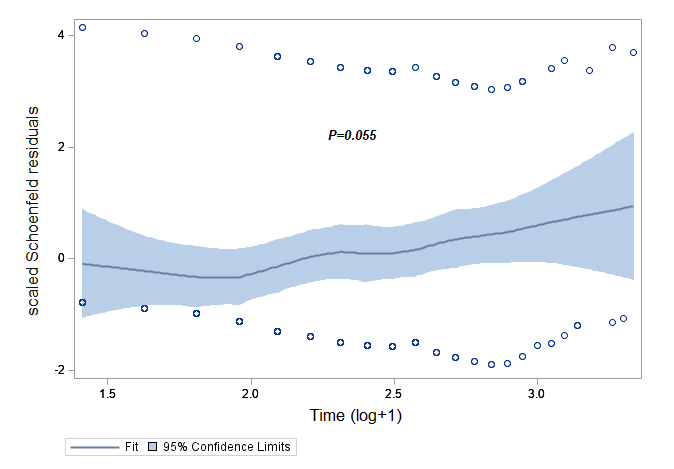
^

**e-figure 2: 28-day Cumulative incidence of VAP (A), All-cause mortality (B), Extubation alive (C) and ICU discharge alive (D). Competing risks: A extubation within 28-day (death or alive); C death under mechanical ventilation; D death in ICU.**

Abbreviations: ICU=intensive care unit; VAP=Ventilator-associated pneumonia)

**A)**

**
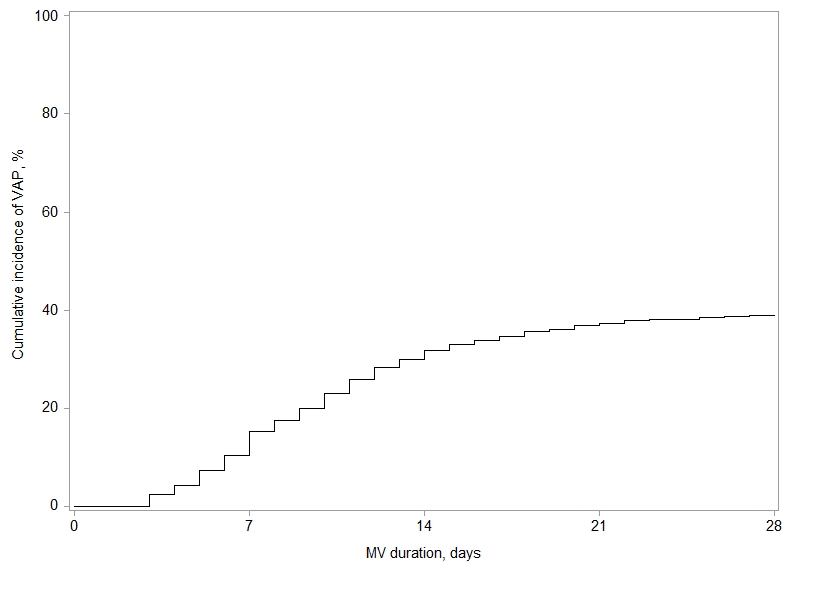
**

**B)**

**
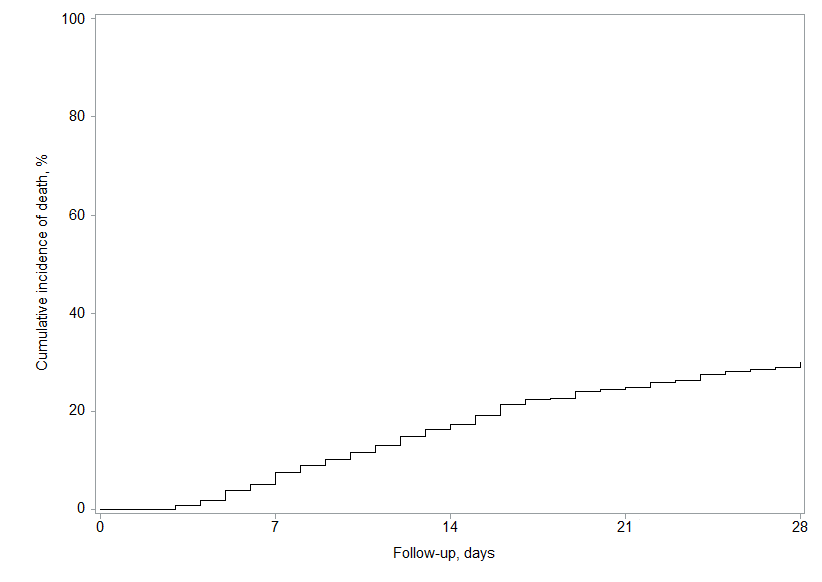
**

**C)**

**
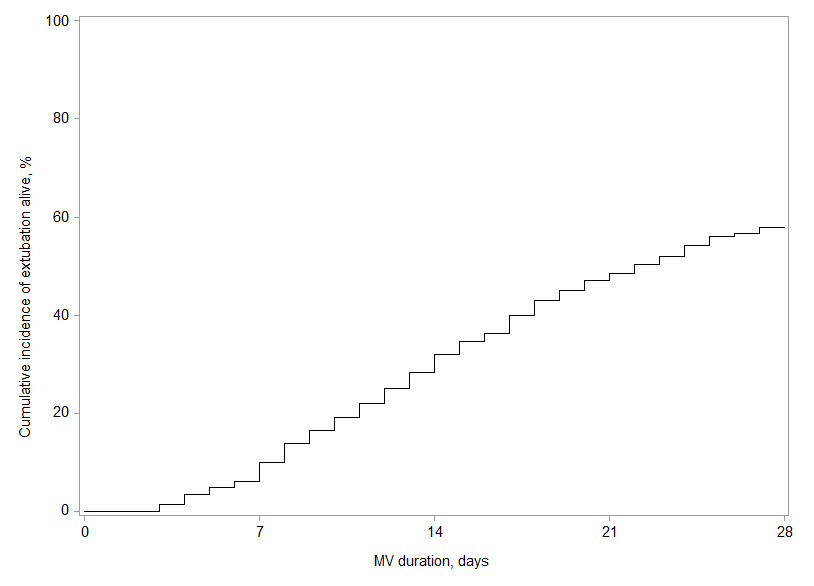
**

**D).**


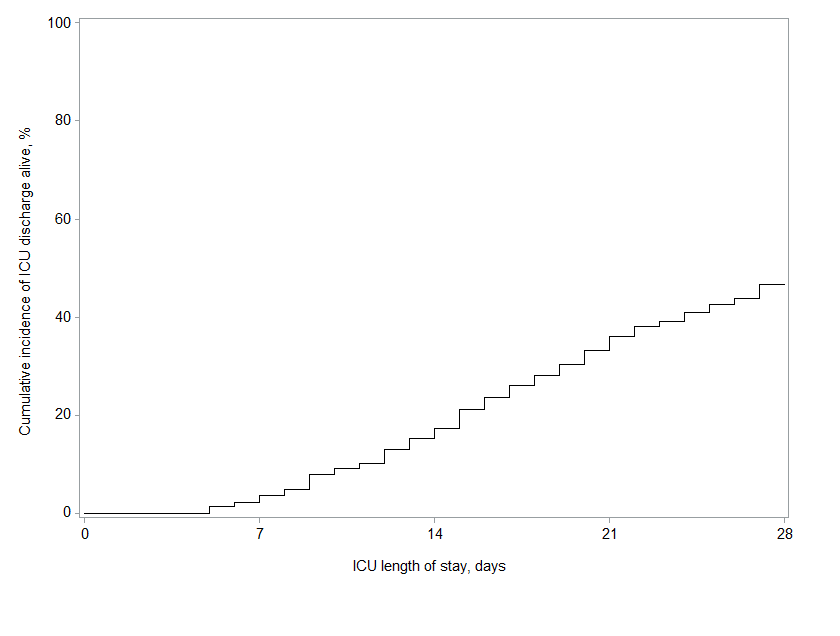


**e-Table 1. Association of the different corticosteroid drugs with VAP, 28-day mortality, MV duration and ICU length of stay in all study patients.**

|  |  | **Unadjusted analysis** | |  | **Adjusted analysis**^2^ | | | | |
| --- | --- | --- | --- | --- | --- | --- | --- | --- | --- |
|  |  |  |  |  | **Multiple imputation analysis^3^** | |  | **Complete case-analysis** | |
| **Corticosteroids** | **Outcomes** | **cHR (95%CI)** | **P-Value** |  | **cHR (95%CI)** | **P-Value** |  | **cHR (95%CI)** | **P-Value** |
| Hydrocortisone vs. none | VAP |  |  |  |  |  |  |  |  |
|  | Overall effect |  | 0.94^1^ |  |  | 0.77 |  |  | 0.62 |
|  | At day 2 | 1.01 (0.22 to 4.56) |  |  | 1.37 (0.29 to 6.36) |  |  | 0.67 (0.11 to 3.80) |  |
|  | At day 7 | 0.93 (0.52 to 1.67) |  |  | 1.25 (0.68 to 2.32) |  |  | 1.10 (0.55 to 2.18) |  |
|  | At day 14 | 0.89 (0.42 to 1.86) |  |  | 1.18 (0.55 to 2.55) |  |  | 1.52 (0.68 to 2.36) |  |
|  | At day 21 | 0.86 (0.28 to 2.60) |  |  | 1.14 (0.37 to 3.53) |  |  | 1.84 (0.55 to 6.06) |  |
|  | 28-day mortality | 3.96 (2.64 to 5.92) | <0.001 |  | 2.50 (1.57 to 3.97) | <0.001 |  | 2.11 (1.29 to 3.45) | 0.003 |
|  | MV duration | 1.04 (0.66 to 1.62) | 0.86 |  | 1.53 (1.07 to 2.15) | 0.017 |  | 1.27 (0.76 to 2.11) | 0.37 |
|  | Length of ICU stay | 0.54 (0.29 to 0.99) | 0.048 |  | 1.44 (1.01 to 2.05) | 0.042 |  | 0.67 (0.34 to 1.34) | 0.26 |
| Dexamethasone vs. none | VAP |  |  |  |  |  |  |  |  |
|  | Overall effect |  | 0.078^1^ |  |  | 0.039^1^ |  |  | 0.049^1^ |
|  | At day 2 | 0.36 (0.06 to 1.83) |  |  | 0.26 (0.04 to 1.55) |  |  | 0.30 (0.04 to 1.83) |  |
|  | At day 7 | 0.99 (0.51 to 1.89) |  |  | 0.94 (0.47 to 1.87) |  |  | 1.04 (0.52 to 2.05) |  |
|  | At day 14 | 1.91 (1.11 to 3.28) |  |  | 2.16 (1.22 to 3.83) |  |  | 2.32 (1.24 to 4.34) |  |
|  | At day 21 | 2.84 (1.21 to 6.65) |  |  | 3.57 (1.42 to 8.99) |  |  | 3.77 (1.37 to 10.31) |  |
|  | 28-day mortality | 1.47 (0.85 to 2.55) | 0.17 |  | 1.58 (0.90 to 2.76) | 0.11 |  | 1.35 (0.72 to 2.52) | 0.34 |
|  | MV duration | 1.11 (0.74 to 1.67) | 0.61 |  | 1.27 (0.90 to 1.80) | 0.17 |  | 1.37 (0.86 to 2.17) | 0.18 |
|  | Length of ICU stay | 0.84 (0.53 to 1.32) | 0.44 |  | 1.06 (0.74 to 1.52) | 0.73 |  | 1.03 (0.62 to 1.69) | 0.91 |
| Methylprednisolone vs. none | VAP |  |  |  |  |  |  |  |  |
|  | Overall effect |  | 0.27^1^ |  |  | 0.30^1^ |  |  | 0.51^1^ |
|  | At day 2 | 0.29 (0.05 to 1.55) |  |  | 0.33 (0.06 to 1.77) |  |  | 0.46 (0.08 to 2.46) |  |
|  | At day 7 | 0.70 (0.35 to 1.38) |  |  | 0.76 (0.38 to 1.50) |  |  | 0.86 (0.43 to 1.70) |  |
|  | At day 14 | 1.23 (0.75 to 2.02) |  |  | 1.30 (0.79 to 2.13) |  |  | 1.29 (0.73 to 2.26) |  |
|  | At day 21 | 1.74 (0.79 to .378) |  |  | 1.79 (0.82 to 3.88) |  |  | 1.65 (0.69 to 3.90) |  |
|  | 28-day mortality | 1.29 (0.79 to 2.08) | 0.30 |  | 1.15 (0.70 to 1.89) | 0.58 |  | 1.00 (0.57 to 1.75) | 1.00 |
|  | MV duration | 1.23 (0.90 to 1.67) | 0.19 |  | 1.10 (0.83 to 1.46) | 0.49 |  | 1.08 (0.73 to 1.57) | 0.71 |
|  | Length of ICU stay | 0.73 (0.50 to 1.04) | 0.079 |  | 0.79 (0.59 to 1.07) | 0.12 |  | 0.69 (0.44 to 1.06) | 0.086 |

^1^ P-Value for effect of corticosteroids assessed by including corticosteroids and time* corticosteroids terms into Cox’s regression model to account the violation of proportional hazard assumption

^2^ Adjusted for age, sex, BMI, SAPS-II, McCabe classification, immunosuppression, recent hospitalization, recent antibiotics, shock, ARDS, cardiac arrest (^3^after handling missing values by multiple imputations (m=20)

Abbreviations: ARDS= acute respiratory distress syndrome; BMI=body mass index; cHR=cause-specific hazard ratio; CI=confidence interval; ICU=intensive care unit; SAPS II= simplified acute physiology score II; VAP= ventilator-associated pneumonia.
